# SARS-CoV-2, Trait Anxiety, and the Microbiome

**DOI:** 10.3389/fpsyt.2021.720082

**Published:** 2021-09-08

**Authors:** Pascal Büttiker, Simon Weissenberger, George B. Stefano, Richard M. Kream, Radek Ptacek

**Affiliations:** ^1^First Faculty of Medicine, Center for Cognitive and Molecular Neuroscience, Charles University in Prague, Prague, Czechia; ^2^Department of Psychology, University of New York in Prague, Prague, Czechia

**Keywords:** anxiety disorders, brain, cognition, COVID-19, default mode network, glutamic acid, immunity, microbiota

## Abstract

During the COVID-19 pandemic, research on the relationships between the virus and its human host has become fundamental to understand this pathology and its effects. Attaining this profound understanding is critical for the effective containment and treatment of infections caused by the virus. In this review, we present some possible mechanisms by which psychopathological symptoms emerge following viral infections of the central nervous system (CNS). These proposed mechanisms are based on microbial communication and the induced priming of microglial antibody activation within the CNS through Toll-like receptor signaling. In this process, chronic microglial activation causes increased glutamate release in virally-altered, high-density neuronal structures, thereby modulating cognitive networks and information integration processes. This modulation, in turn, we suggest, affects the accuracy of sensory integration and connectivity of major control networks, such as the default mode network. The chronic activation of immunological responses and neurochemical shifts toward an elevated glutamate/gamma-aminobutyric acid ratio lead to negative reinforcement learning and suboptimal organismic functioning, for example, maintaining the body in an anxious state, which can later become internalized as trait anxiety. Therefore, we hypothesize that the homeostatic relationship between host, microbiome, and virome, would be decisive in determining the efficiency of subsequent immunological responses, disease susceptibility, and long-term psychopathological effects of diseases that impact the CNS, such as the COVID-19.

## Introduction

During the coronavirus disease 2019 (COVID-19) pandemic caused by the severe acute respiratory syndrome coronavirus 2 (SARS-CoV-2), it has become essential to understand the intricate relationships between the virus and its human host. Achieving this deeper understanding is critical for the development of holistic and precise treatment strategies. Doing so also helps elucidate organismic adaptations and functional mechanisms in an evolutionary meaningful manner. In this regard, the gut microbiome and its various effects on human physiology are being studied extensively. For example, the study of germ-free mice allowed early observations of the effects of the absence or imbalance of bacteria on mammalian phenotypes and genotypes (1, 2). Several *in vivo* and *in vitro* studies have been conducted that helped explain host-microbial interactions in health and disease.

In the present review, we introduce possible mechanisms to explain how inter- and intra-species bacterial and viral communication, directly and indirectly, affects host physiology and the psyche in health and disease. We propose that the mechanisms involve the microenvironmental homeostasis (symbiosis/dysbiosis) of a multi-organismic holobiont (namely, humans) that controls systematic regulation and, therefore, individual functioning (3). Given the growing body of literature concerning COVID-19's toll on mental health, we surmise that recognizing alternate pathways for immune activation and viral interactions is necessary for the comprehension, limitation, and treatment of psychopathological symptoms, which appear to depict a significant role in the long-term effects of SARS-CoV-2 infection (4–7).

**Figure 1:**
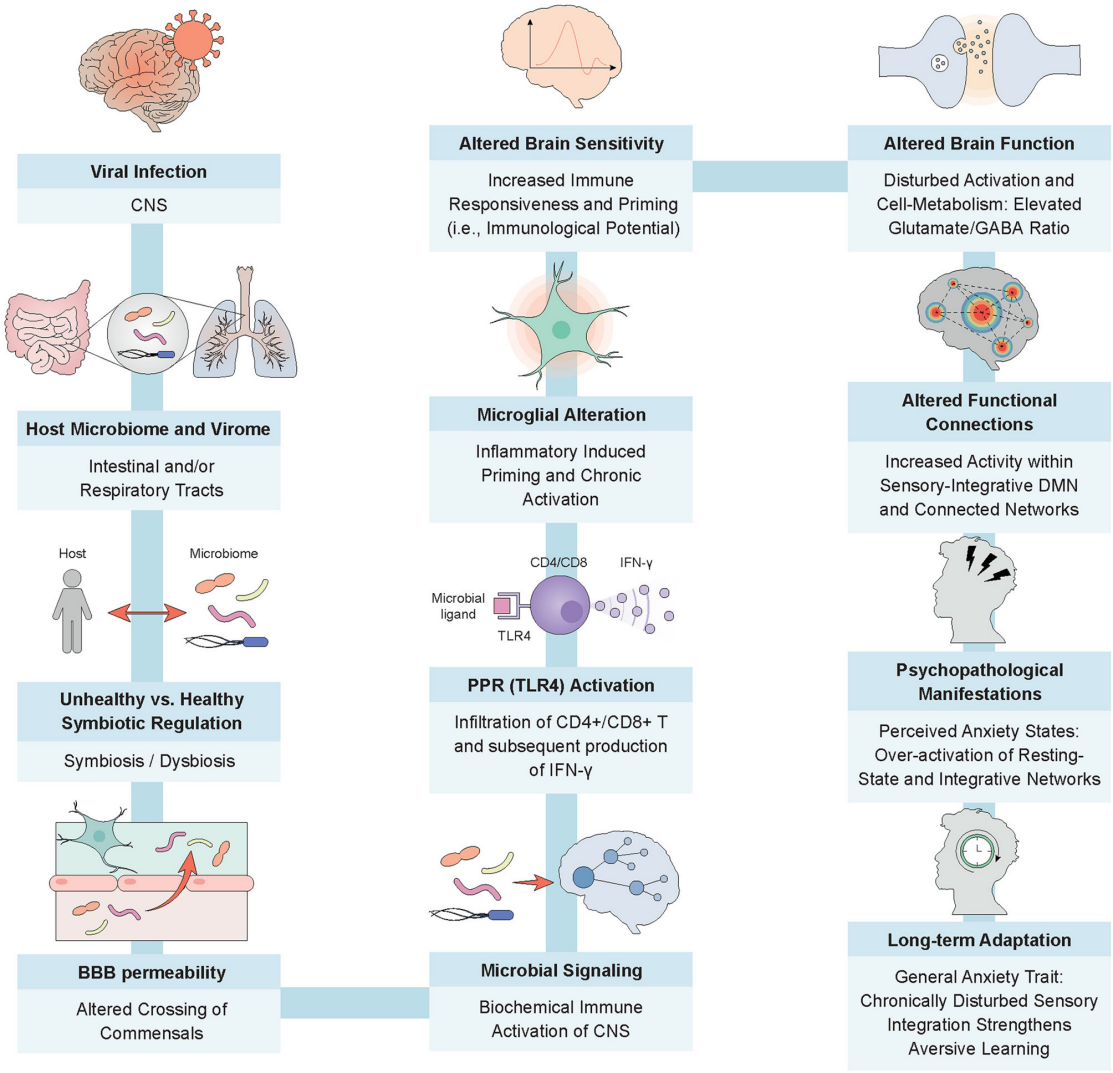
From viral infection to trait anxiety; the intricate processes of a microbially-guided immune response. The model depicts a potential route for the emergence of trait anxiety post SARS-CoV-2 infection. Depending on the host's microbial homeostasis, increased permeability of barriers, for example, gut and blood brain, fosters the migration of commensals and associated immune cells, such as macrophages, into the vasculature (8, 9). Microbial dysbiosis can also promote the crossing of enhanced levels of endotoxic products and proinflammatory stimulating microbes through a disrupted mucosal epithelium, activating TLR4 receptors on dendritic cells. This phenomenon has the potential to facilitate the exchange of neurochemical signals and commensal products through the blood brain barrier, thereby stimulating the infiltration of CD4+ and CD8+ T cells and the subsequent production of IFN-I within the CNS by way of microglial activation (10–12). Enhanced immunocyte entrance into the privileged brain compartment may also generate inflammatory responses in the resident sentinel immune cells, namely microglia. Abnormal microglial priming and successive chronic activation may cause increased glutamate release in virally-altered, high-density neuronal structures, modulating the functional connectivity of brain networks responsible for information integration processes (13–15). Chronic activation of immunological and neurological responses, for example, a disturbed coordination between the excitatory neurotransmitter glutamate and inhibitory neurotransmitter gamma-aminobutyric acid (GABA) in the form of an elevated glutamate/GABA ratio, may lead to insufficient deactivation of major modulatory networks, such as the default mode network, and to over-attentiveness to perceptually integrated stimuli (i.e., rumination) (16). This has the potential to lead to negative reinforcement learning and perceived states of anxiety (17). The consistent subliminal inflammation caused by the persistent virus upholds the microbial dysbiosis, reinforcing the negative cycle of suboptimal organismic functioning and maintaining the individual in this anxious state, which, as we now surmise, later manifests as trait anxiety (17–20).

For this purpose, we provide an empirically based hypothetical model of viral infection (see Figure 1). The model explores possible paths by which a virus induces physical alterations in the internal milieu and generates maladaptive neuronal signaling to the central nervous system (CNS) via microbial, dysbiotic communication. We elaborate on the psychopathology by considering how microglial alterations and subsequent metabolic changes of the neuronal system can cause altered activation patterns of integrative brain networks (21). These alterations may result in recurrent anxiety that becomes internalized as trait anxiety. Finally, we discuss a microbial approach to disease prevention and treatment.

## Microbial Signaling: a Substrate for Proper Immune Functioning

The microbiome plays a critical role in communicating signals that mediate immune responses to foreign intruders (2, 22). Bacteria participate in intra-species communication to initiate neurochemical signaling and conduct inter-species information exchange with fungi and viruses in the form of enzymatic and neurochemical processes (10, 23). Outnumbering the total human cell count, microorganisms are believed to form a highly complex communication network (10).

In viral infections, gastrointestinal and respiratory tract bacteria may play essential roles in communicating appropriate responses (24). By direct and indirect routes, bacteria may interact with the intruder (i.e., virus), alter the permeability of barriers that inhibit migration of commensals into the circulation, or send neurochemical signals to the CNS via enteric nervous system (ENS) connections. In this manner, bacteria may alter viral interactions with the host (10, 25). Depending on the microenvironment composition, the microbiota can either combat viral replication or promote it (10, 24). Whether a microbially-initiated immune response to a viral encounter limits or promotes viral replication may depend on the symbiotic regulation mechanisms between the host and microbiota. Thus, the homeostatic relationship (symbiosis/dysbiosis) between the host and microbiome may be crucial to disease onset (10, 25, 26).

When a virus enters the host via the upper respiratory or gastrointestinal tract, the microbiota and its products are believed to directly interact with the viral intruder, thereby altering responses and infectivity (10, 24). One such mechanism is the microbiota-initiated immune priming through Toll-like receptor (TLR) signaling (25). TLRs belong to a family of pattern recognition receptors that influence systemic immune response (22, 25). The microbiota is rich in microbial ligands that can bind to TLRs, thereby initiating and directly altering immune responses (25).

Especially in case of viruses that can penetrate the CNS, precise non-cytolytic clearance is fundamental (11). Such clearance avoids damage to essential and often non-renewable neuronal cells. Successful non-cytolytic clearance and neutralization of infective viral particles involve regulated infiltration of cytotoxic CD4+ and CD8+ T cells and viral specific antibodies, leading to downstream production of anti-viral type II interferon-γ (IFN-γ) (11). Indeed, microbial activation of TLR ligands can prime microglia in the CNS, upregulating antigen presentation activity (25). The initiation of antiviral responses via TLR-mediated IFN-I secretion by various *Lactobacillus* strains has been extensively studied (22).

The exact signaling pathways used for microbial communication with the CNS remain unclear (25). Under normal physiological conditions (e.g., controlled states of inflammation), the colonic epithelial barrier provides a protective shield, inhibiting the uncontrolled flow of commensals into the circulation (8, 9). This systemic immunity is maintained by homeostatic microbial activation of TLR2 receptors (12). Microbial dysbiosis, however, can lead to disruption of the epithelial barrier, causing proinflammatory microbes to activate TLR4 receptors on dendritic cells, allowing commensals and their products to enter the circulation (12). Despite many commensals and products not directly crossing the blood-brain barrier, dendritic cells in the meninges and cerebrospinal fluid might upregulate antigen presentation within the CNS through lymphatic vessels and joint fluid drains in deep cervical lymph nodes (11, 27). A recent study suggests that diverse microbial metabolic products also exhibit the necessary chemical properties to cross the blood-brain barrier, thereby directly altering brain physiology and functionality, for example, via microglial expression (21, 22).

Another pathway for microbial commensals and their products to affect the CNS is through distant signaling of the ENS via the vagus nerve (VN), the primary avenue of bidirectional gut-brain communication (9). The VN is a feature of the autonomic nervous system that, when infected by microbes, can, directly and indirectly, alter the CNS, for example, by way of parasympathetic over- or under-activation (9). These alterations may lead to immune regulatory effects, intestinal permeability, and changes in neurotransmitter and endocrine balance (22). Hence, microbial dysbiosis can cause physiopathological and psychopathological symptoms such as anxiety, depression, irritable bowel syndrome, and others (22). Psychopathological symptoms may likewise be initiated by bacterially-altered neurotransmitter activity via afferent vagal fibers (9, 22, 28). Neuropods on enteroendocrine cells also support the transmission of signals to the CNS through ENS signaling (25). In sum, SARS-CoV-2 infection of gut endothelial cells destroys normal signaling between intrinsic cells, ENS neurons, and commensal bacterial strains. This leads to a subsequent loss of secreted essential short-chain fatty acids (SCFAs) (12) and to pathogenic bacterial adjuvants, additives or a synergistic combination of gut-derived and blood-derived inflammatory molecules that profoundly affect complex cognitive and affective integrative processes within the CNS.

## Infection of the Brain: Chronicity, Neuronal Activity, and Cognition

Preclinical and clinical studies of well-known neuroinvasive/neurotropic viruses may provide valuable insight into the cellular and molecular mechanisms associated with the deleterious neurological and cognitive sequelae of SARS-CoV-2 infection within CNS structures. For example, a mouse model study has demonstrated chronic cognitive neurological deficits following West Nile virus (WNV) or Zika flavivirus infection (13). The authors demonstrate association of the persistence of infiltrative hippocampal T cells and IFN-γ signaling and spatial-learning defects. Furthermore, neurotropic/neuroinvasive WNV infection has been associated with altered blood-brain barrier (BBB) permeability with a subsequent release of IFN-γ and other proinflammatory cytokines (29). Accordingly, a recent observational clinical study demonstrated increased concentrations of the key anti-viral/proinflammatory cytokines interleukin-6 (IL-6) and IFN-γ in cerebrospinal fluid (CSF) samples of patients with neuroinvasive WNV infection (30). Finally, in a preclinical rodent model of recurrent neuroinvasive HSV-1 infection, expression of neuroinflammatory cytokines, such as IL-1β and IL-6, in the hippocampus of HSV-1 infected mice was correlated with an increased incidence of cognitive deficits (31).

A recent review and critical discussion of SARS-CoV-2-associated neurological, cognitive and psychiatric deficits presents a multi-faceted profile of putative mechanisms underlying the neurotropic/neuroinvasive potential of the virus (32). Complementary *in vivo, in vitro* and neuropathological studies suggest convergent and potentially synergistic strategies used by SARS-CoV-2 for neuroinvasion, infection, and replication within CNS neurons and astrocytes with subsequent cellular and tissue damage linked to chronic neurological dysfunction (33–35). For example, Song and colleagues utilized a three-pronged approach to probe potential mechanisms of SARS-CoV-2 neurotropism *via in vitro* usage of human brain organoids, *in vivo* employment of transgenic mice overexpressing human ACE2, and analysis of human autopsy specimens from patients who died from COVID-19-associated pathologies. Overall, the authors provided evidence for the neuroinvasive capacity of SARS-CoV-2 in an apparent ACE2-dependent mechanism. In contrast, the lack of consistency of recent *in vitro* and *in vivo* datasets supporting a direct neuroinvasion by SARS-CoV-2, suggests that a virus-induced loss of BBB integrity with heightened CNS influx of inflammatory molecules is the likely culprit in long COVID-associated neurological and psychiatric disorders (32). Accordingly, these long-term debilitating neurological sequelae appear to be functionally linked to chronic inflammatory conditions within CNS structures and potential key entry points, such as choroid plexa (18), *via* chronically activated microglia.

Recent research suggests that viral infections of the CNS target the mitochondrial system and RNA components, hijacking the host's cellular energy structure and genetic blueprint (35, 36). It is believed that SARS-CoV-2, too, functionally diverts a significant portion of the cellular bioenergetics capacity to support replication of morphologically and chemically competent viral particles effectively, and that these mechanisms possibly supervene cognitive symptoms (36). This hypothesis is also supported by evolutionary host-microbe interactions in the process of natural selection, where prokaryotes, eukaryotes, and viruses try to form symbiotic relationships with a host to ensure/prolong their respective lineages (3, 37). In this manner, the microbes may affect genotype and phenotype over the long term (3, 37). These interactions may compromise the host resulting in suboptimal functioning and possible disease-related symptoms (10).

Given the nature of CNS viruses that infiltrate and hijack energy-generating systems for replication and survival, they are predicted to infect localized, high neuronal-density networks in the brain during disease onset (35). Control networks are characterized by vast interconnections with lower localized neuronal density; therefore, they may be less affected initially (14, 15, 38, 39). Apoptosis or neuronal metabolic alterations caused by virally-induced inflammation/infiltration and microglial activation may be responsible for disproportional functional connectivity between neighboring brain networks (17).

We hypothesize that functional disturbances initially may affect mainly high-density localized brain networks such as primary sensory cortices, leaving lower-density interconnected control networks largely intact, thereby causing alterations in local sensory processing and integration into higher-order control networks (15).

In a predictive coding model, a virally unaffected integrative control network would display higher functional reliance and connective accuracy than networks affected by infections, possibly causing a shift to higher-order-based functionality and alteration in resting-state functional connectivity (16, 40). Given recent research on the impact of higher-order control network overconnectivity, specifically of the default mode network (DMN), these effects may explain several mental and physical symptoms (41, 42).

Functional and structural brain alterations may be initiated directly by microbial signaling (i.e., TLR binding by bacterial antitoxins) (22, 43). Through subsequent priming and activation of microglia in affected brain regions (i.e., activation by IFN-γ), metabolic processes and neurochemical release can be altered (22, 43). Given the critical role of the microglia in innate immunity signaling, with varying levels during activation and rest, microglial priming can directly modify essential brain functions (43). Chronic microglial activation, for example, causes increased responsivity to immune stimulation during non-stimulated conditions as well, leading to heightened immune responses due to chronically elevated baseline cytokine levels (43). This phenomenon modifies phenotypes toward a more vigilant state due to locally increased glutamate release (43). Virally infected regions with primed microglia may therefore experience a disruption in the form of glutamate-induced overactivation and exaggerated responses (43).

We suggest that microglial priming by a persistent virus acts by preserving chronic cytokine-mediated glutamate release, maintaining the body in a constant state of clearance (43). Moreover, the glutamate overflow and subsequent excitotoxicity lead to an imbalance of the ratio between synaptic and extracellular glutamate and gamma-aminobutyric acid (GABA) (44). This glutamate-dominant imbalance curbs the synaptic expression of GABA, thereby promoting overexcitability and maladaptive signal processing of neuronal systems and brain networks (44).

During an infection, a glutamate-favored ratio is caused by high levels of inflammatory-induced cytokines and subsequent glutamate release that leads to a spillover of synaptic glutamate into the extra-synaptic space (43). A decreased capacity to clear the extracellular glutamate promotes glutamate binding to activated microglial receptors (i.e., ionotropic and metabotropic). This further enhances the production of inflammatory cytokines and the subsequent release of glutamate and nitric oxide into the extracellular space (43, 45). The resulting overactivation of ionotropic receptors (i.e., NMDA) and nitric oxide synthase-mediated oxidative stress causes alterations in cell metabolism and cytotoxicity (45). The alternating concentration of synaptic and extra-synaptic glutamate and successive shifts in ionotropic receptor activation induce synaptic uncertainty and unstable neurotransmission, possibly undermining inhibitory activity (43, 44). Therefore, these mechanisms may be responsible for mental health issues associated with viral infections of the CNS (7, 43, 44).

A direct link between virally- or bacterially-induced microglial activation/priming and mood disorders has been established. For example, overactivation of microglia in the anterior cingulate cortex can be found in patients with depression (43). It is believed that this phenomenon is in part due to the mediating role of the glial cells in controlling inflammation and glutamate release that affect behavior (i.e., arousal, fear, anxiety) as well as also long-term plasticity (i.e., learning) through altered reward evaluation (43). Thus, the increased local exaggerated release of the neurotransmitter glutamate can cause systemic overactivation of critical primary sensory systems and higher-order networks. This phenomenon may give rise to excessive integration of sensory information and increased rumination in higher-order control networks, including the DMN, thereby leading to neuropsychiatric symptoms (46, 47).

## The DMN: Persistent Infection, Aversive Learning, and Anxiety

Effective inter-network communication is essential for successfully integrating information into higher-order mental processes such as emotion regulation and decision-making (16, 19, 20). The fine-tuned interplay between various functional networks facilitates attention, control, and integration of environmental changes (19, 48).

The DMN is a functionally and structurally highly interconnected brain network involved in tasks such as mentalizing, autobiographical memory, spontaneous cognition, self-referential processing, and high-level aspects of emotion (49). A generally lower density of neuronal cell bodies and increased density of dendric spines confirms its interlinked nature for inter-modular associative processing (15). The DMN shows increased activity, especially during non-goal-directed cognitive tasks. The inactivation (i.e., suppression) of the DMN is associated with exogenous activity, whereas its activation supports endogenous activity (47). By regulating attention/disattention, the DMN participates critically in constructing normal and abnormal learning and perception (47, 50). Abnormal DMN activation and altered within-network functional activity patterns have been connected to various neuropsychiatric disorders, including schizophrenia, anxiety, and depression (47, 50, 51). Disturbed neurochemical coordination between the excitatory neurotransmitter glutamate and inhibitory neurotransmitter GABA, for example, can lead to insufficient DMN deactivation and, therefore, over-attentiveness to perceptually integrated stimuli (i.e., rumination) (47). Specifically, increased functional connectivity and altered intrinsic activity within the core region of the DMN, the posterior cingulate cortex, has been associated with depression, anxiety, and states of worry. In general anxiety disorders, this increased functional connectivity between DMN regions is believed to cause enhancement in sensory processing and flawed integration of environmental stimuli, giving rise to general tension and vigilance in the resting state as well (41).

In summary, the chronic microglial-primed glutaminergic overactivation, inflammatory-induced systemic parasympathetic deactivation, and stress-associated endocrine adaptations that result from microbial and viral communication form an ideal environment for maladaptive perception and learning.

We suggest the danger in acquiring virus-induced long-term symptoms may lay in the long-lasting effects of neuroadaptation. If viral CNS infiltration is not restricted in the early stages of infection, functional and structural changes may spread from high-density to lower-density brain networks. Subsequent changes in neuronal activity and connectivity due to localized, hypoxic apoptosis, inflammatory-forced neuronal metabolism, and altered neurochemistry might cause disturbed sensory prediction and behavioral disruption (7, 16). Chronic high sensitivity to sensory integration, unsuccessful filtering of information, and high-level rumination of possibly distorted concepts may foster negative reinforcement learning (46). This sequence can directly strengthen uncertainty, alertness, and emotionally aversive prior expectations through long-term potentiation (46, 52).

Furthermore, the generally heightened threat expectations due to the chronic immunological priming of microglia by a lingering virus increase the body's readiness to infection, maintaining the individual in an energy-intensive state of alertness (16, 53). Consistent activation of immunological responses may cause chronic inflammation and microbial dysbiosis, reinforcing the negative cycle of suboptimal organismic functioning and maintaining the individual in an anxious state to the point where the anxiety is internalized and becomes a trait (16, 54, 55).

In a predictive coding model of the mind, the heightened immunological potential and subsequently induced responses can foster aversive learning by conceptual overgeneralization (16, 46). Aversive affect, such as occurs following the recurrent experience of anxiety states, may be elicited by minimizing inflammatory-based prediction errors in the form of active interoceptive inference (e.g., sympathetic/parasympathetic nervous system alteration). Because these virally-induced anxiety states are not inferable to a distinct consciously perceived sensory cause, the host may convey the aversion to other, non-aversive components of higher-order concepts (16, 56). Hence, the subsequent overgeneralization of negative interoceptive responses to neutral sensory evidence can become funneled into a wide array of conceptual components, giving rise to persistent uncertainty and generalized anxiety (16, 46).

## Microbial Symbiosis: Disease Susceptibility, Prevention, and Treatment

Given the microbial communication-dependence of initiation of primary and secondary immune responses, the symbiotic relationship between microbial entities and their hosts may be considered a significant control panel for the switching between health and disease. Therefore, we suggest that individuals may differ substantially in terms of susceptibility to deep-structure viral infiltration and symptom severity depending on their pre-infectious microbial homeostasis, immune defense strategies, and neuro-physiological environment, resulting in varying long-term alterations (i.e., learning).

Especially during the COVID-19 pandemic, the question of microbial treatment should be addressed. Discussions have increasingly focused on probiotic bacteria for the prevention, control, and treatment of viral infections. Given the diverse immune-modulatory effects of commensal communication, promoting a symbiotic relationship between the microbiome and the host appears to be a natural step toward disease prevention. It has been suggested that probiotics can reduce susceptibility to viral infections and the severity of disease progression (57). These effects may be mediated by generally enhanced immune activity, possibly allowing early detection and containment of the intruder through effective targeting and enhanced CNS signaling. Maintaining a healthy microbial symbiosis may be critical to successful systemic reactions and interactions (58, 59).

Concerning microglial priming by virally- or bacterially-induced inflammation and the successive overflow of synaptic and extra-synaptic glutamate, the use of probiotics shows promising results. Experimental data suggest that the administration of concentrated lactobacilli- and bifido-bacteria may ameliorate artificially-induced glutamate excitotoxicity by increasing depleted GABA (44). We recommend that further investigation is required to determine the antiviral potential of artificially-induced microbial alterations.

## Conclusion

We suggest possible mechanisms by which psychopathological symptoms emerge following viral infections that impact the CNS. By way of microbial communication and subsequent alterations of the internal milieu, commensals can directly and indirectly promote or limit viral replication in humans. Through various communication routes, microbial products can prime microglial antibody activation within the CNS through TLR signaling, thereby stimulating the infiltration of CD4+ and CD8+ T cells and the subsequent production of IFN-I. Microglial priming and chronic activation may cause increased glutamate release in virally-altered, high-density neuronal structures, modulating cognitive networks and information integration processes. Chronic activation of immunological responses and neurochemical imbalance in the form of an elevated glutamate/GABA ratio may lead to negative reinforcement learning and suboptimal organismic functioning, maintaining the body in an anxious state, which may later become internalized as trait anxiety. This is a possible explanation for developing anxiety post infections, emphasizing the importance of the microbiome/virome connection in mental and physical health (which, in our view, are identical). Therefore, we hypothesize that the homeostatic relationship between host, microbiome, and virome is decisive in determining the efficiency of subsequent immunological responses. Further research concerning the use of probiotic bacteria for the prevention and treatment of viral infections is recommended.

## Author Contributions

PB conceived the presented idea. PB, SW, and GS wrote the manuscript. GS and RK provided the neurobiological background. GS and RK produced the figure and figure legend. RP supervised the project. All authors contributed to the article and approved the submitted version.

## Funding

This work was supported by Charles University, Prague (Progres Q 06 1. LF. UK.).

## Conflict of Interest

The authors declare that the research was conducted in the absence of any commercial or financial relationships that could be construed as a potential conflict of interest.

## Publisher's Note

All claims expressed in this article are solely those of the authors and do not necessarily represent those of their affiliated organizations, or those of the publisher, the editors and the reviewers. Any product that may be evaluated in this article, or claim that may be made by its manufacturer, is not guaranteed or endorsed by the publisher.
